# Biopolymer Extracted from *Anadenanthera colubrina* (Red Angico Gum) Exerts Therapeutic Potential in Mice: Antidiarrheal Activity and Safety Assessment

**DOI:** 10.3390/ph13010017

**Published:** 2020-01-18

**Authors:** Thiago S. L. Araújo, Taiane M. de Oliveira, Nayara A. de Sousa, Luan K.M. Souza, Francisca B. M. Sousa, Ana P. de Oliveira, Lucas A. D. Nicolau, Alfredo A. V. da Silva, Alyne R. Araújo, Pedro J. C. Magalhães, Daniel F. P. Vasconcelos, Hugo R. de Jonge, Marcellus H. L. P. Souza, Durcilene A. Silva, Regina C. M. Paula, Jand Venes R. Medeiros

**Affiliations:** 1 The Northeast Biotechnology Network (RENORBIO), Federal University of Piauí, Teresina, PI 64049-550, Brazil; thiago_parnaiba@hotmail.com (T.S.L.A.); nayaranayvinsck@hotmail.com (N.A.d.S.); apatriciabiomed@gmail.com (A.P.d.O.); vasconcelos@ufpi.edu.br (D.F.P.V.); durcileneas@gmail.com (D.A.S.); 2 Biotechnology and Biodiversity Center Research, BIOTEC, Federal University of the Parnaíba Delta, Parnaíba, PI 64202-020, Brazil; taianeoliveiraphb@gmail.com (T.M.d.O.);; 3 Department of Physiology and Pharmacology, Federal University of Ceará, Fortaleza, CE 60430-275, Brazil; lucasnicolau5@hotmail.com (L.A.D.N.); alfredoaugvs@gmail.com (A.A.V.d.S.); pjcmagal@ufc.br (P.J.C.M.); souzamar.ufc@gmail.com (M.H.L.P.S.); 4 Department of Gastroenterology & Hepatology, Erasmus University Medical Center, 3000 CA Rotterdam, The Netherlands; h.dejonge@erasmusmc.nl; 5 Department of Organic and Inorganic Chemistry, Federal University of Ceará, Fortaleza, CE 60451-970, Brazil; rpaula@dqoi.ufc.br

**Keywords:** polysaccharide, Fabaceae, diarrhea, cholera, *Escherichia coli*

## Abstract

*Anadenanthera colubrina* var. cebil (Griseb.) Altschul (Fabaceae family), commonly known as the red angico tree, is a medicinal plant found throughout Brazil’s semi-arid area. In this study, a chemical analysis was performed to investigate the antidiarrheal activity and safety profile of red angico gum (RAG), a biopolymer extracted from the trunk exudate of *A. colubrina*. Upon FT-IR spectroscopy, RAG showed bands in the regions of 1608 cm^−1^, 1368 cm^−1^, and 1029 cm^−1^, which relate to the vibration of O–H water molecules, deformation vibration of C-O bands, and vibration of the polysaccharide C-O band, respectively, all of which are relevant to glycosidic bonds. The peak molar mass of RAG was 1.89 × 10^5^ g/mol, with the zeta potential indicating electronegativity. RAG demonstrated high yield and solubility with a low degree of impurity. Pre-treatment with RAG reduced the total diarrheal stool and enteropooling. RAG also enhanced Na+/K+-ATPase activity and reduced gastrointestinal transit, and thereby inhibited intestinal smooth muscle contractions. Enzyme-Linked Immunosorbent Assay (ELISA) demonstrated that RAG can interact with GM1 receptors and can also reduce *E. coli*-induced diarrhea in vivo. Moreover, RAG did not induce any signs of toxicity in mice. These results suggest that RAG is a possible candidate for the treatment of diarrheal diseases.

## 1. Introduction

Since ancient times, the plant kingdom has been an important source for the discovery of new drugs, and several therapeutic agents have been isolated from a variety of plant species [1,2,3,4,5,6]. The document “Strategies on Traditional Medicine” from the World Health Organization (WHO), promotes the strengthening of quality assurance, safety, and proper use of medicinal plants, which suggests the regulation of products and practices associated with plants used in folk medicine [7]. This is a necessity because the natural products derived from plant species may not contain the active component in sufficient quantities, due to environmental influences. Additionally, the identity of the active compound is often unknown [8]. This may be a limitation since it is known that molecular weight, charge density, solubility, and/or elementary distribution all play an important role in the possible biological activities of these products [9]. Thus, although the population uses several plant-derived products due to their therapeutic properties, obtaining purified compounds for high-precision identification is still necessary to further characterize their composition [10]. 

One plant with great cultural and medicinal value is *Anadenanthera colubrina* var cebil (Griseb.) Altschul, a plant from the Fabaceae family. In particular, *A. colubrina* is considered one of the most essential medicinal plants of the Caatinga and Cerrado area (the Brazilian semi-arid ecosystems), where it is popularly known as the red angico tree [11,12]. *A. colubrina* stands out due its medicinal properties, which include the application for treatment of inflammatory disorders in different tissues [13,14]. Several studies have shown the various pharmacological properties of products derived from *A. colubrina* (mostly extracts and fractions from the leaves and roots) that include antimicrobial [12,15,16], antioxidant [17,18,19,20], wound-healing [21,22], anti-inflammatory [23], antinociceptive [24], and antiproliferative properties [18,25]. Despite the therapeutic potential, little information is available regarding the chemical properties and biological activities of the biopolymer extracted from the trunk exudate of *A. colubrina*, the red angico gum (RAG), of northeast Brazil. Previous studies [26,27,28] have reported that high performance liquid chromatography’s composition of RAG was high in arabinose (67.8%), followed by galactose (24.1%), and a small amount of rhamnose (2.0%), being a high arabinose heterosaccharide with a main chain composed of β- D-Galp, also including α-L-Araf and β-L-Arap [26]. Ethnopharmacological studies show that RAG is widely used in folk medicine. Lorenzi [29] reported that this exudate has been consumed since ancient times by the locals, and water-soluble preparations are commonly used as a pectoral emollient, in broncho-pulmonary disorders, including cough, bronchitis, asthma, and pharyngitis, thereby facilitating sputum expulsion. This same study also describes the use of the RAG exudate for the treatment of gastrointestinal diseases, including diarrhea. 

Natural gums are polysaccharides obtained from the exudates of tree trunks, seeds, algae, or by microbiological fermentation. They tend to hydrate in cold or hot water, forming colloidal dispersions, highly viscous solutions, or even gels and have numerous technological and medicinal applications [30]. The polysaccharides extracted have potential to be used as novel bioactive products for therapeutic treatments. Previous studies performed by our research group have shown beneficial effects alleviating gastro-intestinal complaints, such as naproxen-induced gastric ulcers [31] and diarrhea [32], by using these exudates. Furthermore, polysaccharides are not absorbed by the intestinal mucosa, remaining there for long periods without producing systemic effects, providing a substantial advantage in comparison to the majority of standard drugs [33]. However, there is still a lack of evidence-based studies on the effects of RAG in diarrhea, as well as its mechanism of action that justify the use of red angico tree exudate in folk medicine by some local communities of northeastern Brazil.

Diarrhea is a common symptom associated with gastrointestinal disorders and characterized by an increased frequency of defecation (three or more times per day), increased fluidity of the feces, and/or the presence of blood and mucus, which can lead to water and electrolyte imbalance [34]. In the majority of developing countries, diarrhea remains one of the most common causes of hospitalization, morbidity, and mortality, mainly among children less than five years of age, worldwide [35]. Generally, in developing countries, infectious diarrhea, including enterotoxin-producing bacteria (*Vibrio cholerae* and *Escherichia coli*), parasites (*Entamoeba histolytica*), and viruses (Rotavirus) are the major causes of this clinical manifestation. The transmission of these infectious agents occurs through physical contact or contaminated water and food, and treatment with oral rehydration solutions (ORS) are the first line therapy for diarrhea worldwide [36]. However, currently available ORS are unable to reduce the duration and severity of diarrhea [37]. 

Considering the biological activity of polysaccharides obtained from closely related tree trunk exudates and the diarrhea pathophysiology [32], evaluation of the antidiarrheal action of RAG, the polysaccharide extracted from the trunk exudate of *A. colubrina* (RAG) is highly relevant. The results obtained could potentially lead to the adoption of new therapies and prophylactic measures.

Therefore, a major aim of the present work was to perform a chemical and biological analysis of the biopolymer extracted from the trunk exudate of *A. colubrina*, RAG. We additionally investigated whether RAG has antidiarrheal activity in experimental mouse models induced by castor oil, cholera toxin, and *E. coli* and conducted a safety assessment to determine if RAG can be used as an effective natural medication in endemic areas.

## 2. Results and Discussion

### 2.1. Chemical Analysis of RAG

**Figure 1:**
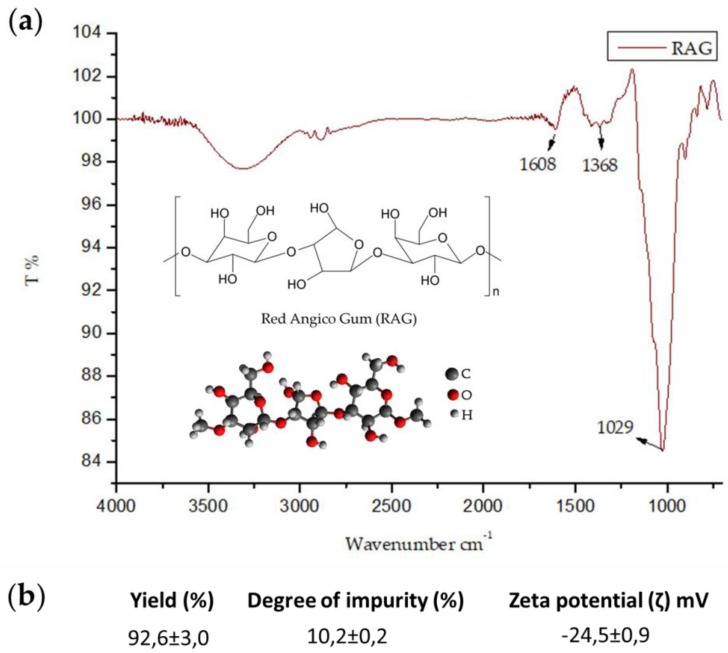
(**a**) FT-IR spectrum of the polysaccharide extracted from the trunk exudate of the red angico tree (RAG) recorded between 700 and 4000 cm^−1^ in attenuated total reflection (ATR) (**b**) Results of yield, degree of impurity, and Zeta potential (ζ) value.

The FT-IR spectra of RAG are shown in Figure 1a. All the IR spectra exhibited absorption bands characteristic of polysaccharides between 4000 cm^−1^ and 700 cm^−1^. With the analysis of the spectra, we observed bands in the region of 1608 cm^−1^ regarding the vibration of O–H water molecules, 1368 cm^-1^ the deformation vibration of C-O bands related to glycosidic bonds, 1080 cm^−1^ and 1029 cm^−1^ related to the stretching of C– O–C and bending of O–H groups present in the glycosidic units. Such results are consistent with those obtained by Sousa et al. [38] for isolated angico gum.

Additionally RAG demonstrated an approximately 93% yield with a low degree of impurity (~10%), suggesting that the trunk exudate of the red angico tree is mostly composed of polysaccharide (Figure 1b). 

The zeta potential reflects the effective charge of the particles and refers to the electrostatic repulsion between them [39]. This analysis showed a negative zeta value (~24 mV) (Figure 1b). Similar loads have been reported by Sousa et al. [38] for isolated angico gum and Quelemes et al. [40] and Silva et al. [41] for cashew gum. 

RAG presented a molar mass of 1.89 × 10^5^ g/mol. The molecular weight of RAG was determined by size exclusion chromatography using molecular weight distribution characterization markers, Mn (number-average molar mass), Mw (mass-average molar mass) and Đ (polydispersity) calculated by the quotient Mw/Mn (Table 1). During the process of purification of the material, it is of extreme importance that the treatments applied to the polysaccharide do not mischaracterize it, guaranteeing intact maintenance of its structure and/or average molar mass. A similar molar mass value was also recorded by De Paula et al. [42]. These results show that RAG is dimensionally larger than polysaccharides extracted from the trunk exudates of other species, such as cashew gum (molar mass of 2.35 × 10^4^ g/mol), obtained from *Anacardium occidentale* L., which is chemically and structurally similar to RAG [41,43]. 

The results of elemental analysis for RAG are presented in Table 1 as well. A discrete amount of nitrogen (0.47%) in RAG may be related to traces of proteins, as has been described by De Paula, Budd and Rodrigues [42] and Silva et al. [26]. These results are consistent with those described in the literature about the elemental composition of isolated angico gum and cashew gum [38,40].

Additionally, the determination of the solubility of RAG at different pHs was performed, and the results are provided in Table 1. RAG showed high solubility at all pH values tested, especially in acidic and basic media, corresponding with the pH found in certain regions in the gastrointestinal tract such as the stomach and the intestines [44], the main sites of action of polysaccharides tested by our research group [31,32,45,46,47]. The structure of RAG is rich in hydroxyl (OH) groups (Figure 1a), which provides a high degree of hydration leading to greater biopolymer interaction with water and consequently greater solubility [48]. 

### 2.2. Antidiarrheal Activity of RAG

#### 2.2.1. RAG Reduces Castor Oil-Induced Diarrhea

The results obtained in our experiments show that the administration of castor oil significantly induced diarrhea and pretreatment with RAG at all tested doses (30, 60, and 120 mg/kg), significantly (*p* < 0.05) reduced total fecal mass (3.92 ± 0.02 g, 28.1%, 2.44 ± 0.02 g, 55.3%, 2.14 ± 0.01 g, 60.8%, respectively) compared to the control group (5.45 ± 0.02 g), 3 hours after administration of castor oil (Table 2).

Castor oil is an effective laxative extracted from the seeds of *Ricinus communis* (Euphorbiaceae) widely used for the screening of drugs with antidiarrheal properties [49], because its decreases fluid absorption, increases electrolyte secretion, and alters intestinal motility [50]. The laxative effect of this oil is attributed to its active principle, ricinoleic acid, an irritant of the intestinal mucosa, which promotes the secretion of endogenous prostaglandins, nitric oxide, platelet activating factor, tachykinins, and cAMP intracellular accumulation [51,52,53,54,55]. 

**Figure 2:**
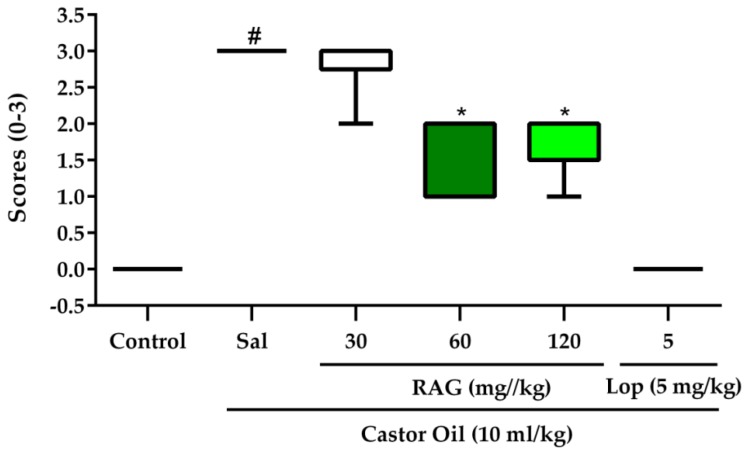
Effects of RAG on the fluidity of feces in castor oil-induced diarrhea in mice. Pretreatment with RAG (30 mg/kg) by gavage did not yield statistically different results compared to the saline+castor oil treatment. However, RAG doses of 60 and 120 mg/kg reduced diarrhea scores and decreased the severity of diarrheal defecation when compared to the control treatment. The standard antidiarrheal drug loperamide (Lop, 5 mg/kg) decreased the production of diarrheal stool significantly. Data are presented as the median and minimum/maximum range. # *p* < 0.01 vs. saline group; * *p* < 0.05 vs. control group. Nonparametric Kruskal–Wallis with Dunn post-hoc test was used for multiple comparisons. Abbreviations: RAG: red angico gum; Sal: saline; Lop: loperamide.

This experiment demonstrated that RAG reduced the total amount of stool (frequency of defecation), especially diarrheal stool (frequency of diarrhea), thereby controlling diarrhea (by 33.7%, 61.2%, and 73.3%, respectively) when compared to the control (Table 2). In addition, RAG at an oral dose of 60 and 120 mg/kg significantly (*p* < 0.05) reduced the severity of diarrhea, as measured by the general diarrhea score and hard, mild, and copious stools, compared to the group treated with saline + castor oil (Figure 2). The standard antidiarrheal drug, loperamide (5 mg/kg, p.o.), inhibited the diarrheal parameters examined with a higher efficacy (*p* < 0.001). 

It can then be inferred that the inhibitory effects of RAG on castor oil-induced diarrhea could be due to inhibition of any of these mediators [56], which would affect the intraluminal fluid accumulation produced by castor oil. Studies by Araújo et al. [32], Sousa et al. [46], Leódido et al. [47], and Bezerra et al. [57] on the antidiarrheal actions of red algae-sulfated polysaccharides of *G. cervicornis*, *H. musciformis*, *G. intermedia*, and a heteropolysaccharide from cashew gum (*Anacardium occidentale*), respectively, showed a result similar to ours, using a similar technique. Our results showed a small difference in effect between the doses of 60 and 120 mg/kg, indicating a plateau effect towards 120 mg/kg. Therefore, the lowest effective dose was used as the standard for further experiments.

#### 2.2.2. RAG Reduces Castor Oil-Induced Intestinal Fluid Accumulation (Enteropooling)

The enteropooling assay was used to evaluate the quantities of intestinal fluid generated by the diarrheal process. Treatment with RAG (30 mg/kg) did not reduce the volume of intestinal contents significantly (10.5%) when compared to the control group treated with saline. However, RAG (60 and 120 mg/kg) significantly (*p* < 0.05) reduced the intestinal fluid volume (56.7% and 59.4%, respectively) compared to the control treatment. Loperamide reduced the fluid volume by 43.6%, (Table 3). This anti-enteropooling effect was better than reported in previous studies with heteropolysaccharide extracts from of *Anacardium occidentale* L. exudate in rats at the same doses [32]. 

#### 2.2.3. Effects of RAG in PGE2-Induced Diarrhea

Another model for the evaluation of diarrhea uses prostaglandin E_2_ (PGE_2_) induction. Prostaglandins contribute to the pathophysiological functions in the gastrointestinal tract with the E type of prostaglandins (PGE_2_) causing diarrhea in experimental animal models as well as humans, owing to dual effects: increasing the gastrointestinal motility and altering the water and electrolyte transport in enterocytes [58,59]. Our results show that PGE_2_ significantly increased the intestinal fluid volume (*p* < 0.001), compared to treatment with saline only (0.73 ± 0.06 mL vs. 0.34 ± 0.07 mL, respectively) (Table 4). However, compared to the PGE_2_ group, the RAG group did not demonstrate a significant reduction in the mean intestinal fluid volume. In the literature, no studies with RAG showing anti-inflammatory activity in systemic acute inflammation models were found. Besides that, assessing RAG involvement in PGE_2_ mediated enteropooling has not been previously explored. We attribute the ineffectiveness of RAG in reducing the intestinal fluid volume, with a few reasons, including evidence that RAG is dimensionally large, as reflected by its molecular weight, thereby making it difficult to bind to PGE_2_ receptors in intestinal mucosal epithelia [60].

#### 2.2.4. RAG Effects on Na^+^/K^+^-ATPase Activity

**Figure 3:**
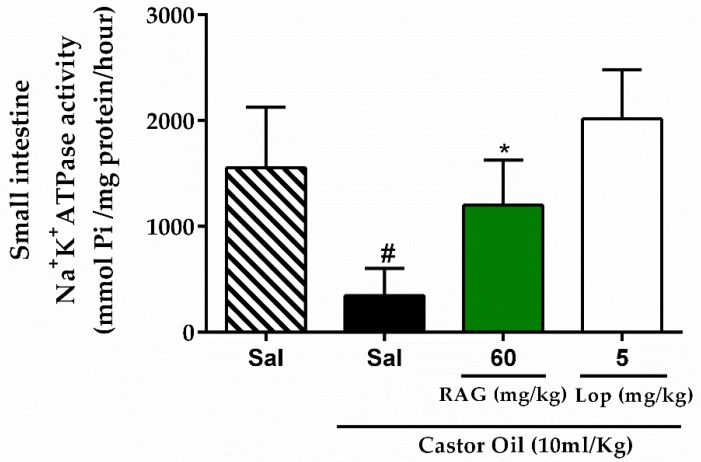
Effect of RAG on Na^+^/K^+^-ATPase activity in castor oil-induced diarrhea in mice. All animals received castor oil (10 mL/kg) by gavage to induce diarrhea. The results are expressed as mean ± SEM. A minimum of six animals were used per group. # *p* < 0.05 vs. saline control group; * *p* < 0.01 vs. saline + castor oil group. Statistics: one-way ANOVA with Tukey post-hoc test. Abbreviations: RAG: red angico gum; Sal: saline; Lop: loperamide.

Studies have shown that castor oil interferes with Na^+^/K^+^-ATPase activity in the intestines, inhibiting its activity and contributing to the fluid accumulation, and the pathophysiology of several diarrheal diseases [61,62]. In this study the results show that castor oil reduced the activity of Na^+^/K^+^-ATPase (345.90 ± 114.09 µmol mg^−1^ h^−1^) significantly (*p* < 0.05) when compared to saline only treatment (1554.00 ± 256.20 µmol mg^−1^ h^−1^) (Figure 3). However the administration of RAG (60 mg/kg) significantly (*p* < 0.05) increased Na^+^/K^+^-ATPase activity in the small intestines (1200.00 ± 191.00 µmol mg^−1^ h^−1^). Thus, we suggest that the increase in the activity Na^+^/K^+^-ATPase in the present study by RAG may contribute to the observed anti-enteropooling effect through a decrease in intestinal fluid accumulation. 

A reduction in the volume and fluidity of stools is usually achieved by reducing both the accumulation of intraluminal water and the loss of electrolytes [55]. Thus, it is likely that RAG increases the reabsorption of electrolytes and water by interfering with Na^+^/K^+^-ATPase activity in the small intestine. Na^+^/K^+^-ATPase is present in the basolateral membrane of the enterocytes and provides the driving force for the active transport of many electrolytes, and inhibiting its activity contributes to the accumulation of fluids [62].

However, this hypothesis does not exclude the possibility of other mechanisms by which RAG may exert its antidiarrheal effect, such as gastrointestinal motility, another physiological mechanism that contributes to diarrhea [63].

#### 2.2.5. RAG Effects on Gastrointestinal Motility

**Figure 4:**
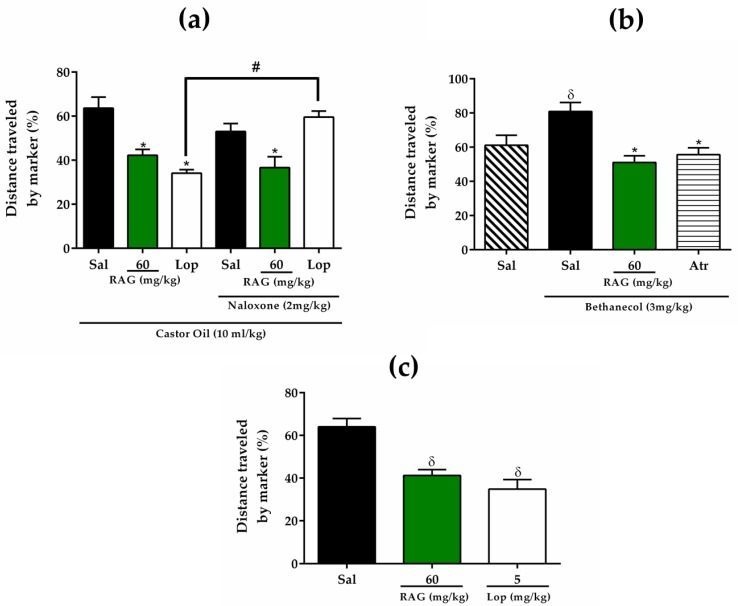
Effect of RAG on intestinal transit in mice. (**a**) Effect of naloxone on RAG activity in castor oil-induced intestinal transit in mice. Pre-treatment with RAG (60 mg/kg, p.o.) significantly reduced (*p* < 0.05) the distance travelled by marker (charcoal) through the small intestine. The treatment with Loperamide (5 mg/kg, p.o.) had a similar anti-motility effect. However, treatment with RAG in the group receiving naloxone (2 mg/kg, s.c.) did not reverse the anti-motility effect of RAG, in contrast to loperamide. * *p* < 0.05 vs. saline group; # *p* < 0.05 loperamide + naloxone vs. loperamide alone. (**b**) Bethanechol, a cholinergic agonist, significantly increased the distance travelled by a charcoal meal through the intestine (*p* < 0.05) compared to the saline treatment group. Pre-treatment with RAG (60 mg/kg, p.o.) reversed the stimulatory effect of bethanechol (3 mg/kg, i.p.). (**c**) In the normal gastrointestinal transit model, pretreatment with RAG (60 mg/kg) showed a significant (*p* < 0.05) inhibition in the gastrointestinal transit of the marker similar to loperamide. These results are expressed as mean ± SEM. Number of animals per group = 5–6. * *p* < 0.05 vs. saline + bethanechol group; δ < 0.05 vs. saline group. Statistics: one-way ANOVA with Tukey post-hoc test. Abbreviations: Lop = loperamide; RAG = red angico gum; Sal = saline; Atr = atropine.

Currently, one of the main therapeutic alternatives for the symptomatic treatment of diarrhea is the use of drugs that act to directly reduce intestinal motility. Among these alternatives, we can highlight the use of loperamide, which inhibits gastrointestinal transit and consequently increases the reabsorption of intestinal fluid by interacting with intestinal opioid receptors [64,65]. In addition to regulating the small intestinal tract, it has also been reported to reduce colon flow rates, thereby affecting colonic motility [66]. In this study, the gastrointestinal distance traveled by a charcoal meal through the intestine after the ingestion of castor oil was used to evaluate the effect of RAG on gastrointestinal transit. Mice treated with RAG or loperamide showed a significant (*p* < 0.01) inhibition in the gastrointestinal transit of the marker (43.661% and 48.225%, respectively), compared to the saline treated group (Figure 4a).

In the second series of experiments, to investigate the possible involvement of opioid receptors on the effect of RAG, we administered a non-specific opioid antagonist. Pretreatment with naloxone did not impair the effects of RAG (60 mg/kg) treatment (30.9% inhibition), although it prevented the activity of loperamide (Figure 4a). Therefore, the antidiarrheal effect of RAG is not dependent on an opioid mechanism and a similar result has been observed with polysaccharides extracted from red algae [45,46,47]. Thus, RAG may delay gastrointestinal transit by other mechanisms that modulate gastrointestinal motility.

In the third series of experiments, a bethanechol-stimulated gastrointestinal transit model was performed. Bethanechol is a cholinomimetic agonist that stimulates intestinal muscle contractility by activating cholinergic muscarinic receptors in the gastrointestinal tract [67]. We found that this cholinergic agonist significantly increased the percentage of distance traveled by a charcoal meal in the intestine (*p* < 0.01) compared to treatment with saline alone (80.9 ± 5.2% vs. 61.1 ± 5.8%, respectively). Mice pretreated with RAG (60 mg/kg) showed a significant (*p* < 0.001) reduction in gastrointestinal transit (36.8%), as did atropine (3 mg/kg, s.c., 31.2% inhibition), a cholinergic antagonist, when compared to the saline-treated group. These results show that RAG reversed the stimulatory effect exerted by bethanechol on gastrointestinal transit (Figure 4b). These results indicate that RAG is able to reduce intestinal motility by blocking the action of muscarinic receptors in intestinal mucosa, acting as an antagonist. 

Finally, our results show that the pretreatment with RAG (60 mg/kg) in the normal gastrointestinal transit model caused significant (*p* < 0.05) inhibition (35.5%), just like loperamide (45.5%) when compared to the saline-treated group (Figure 4c). 

#### 2.2.6. Ex vivo Antispasmodic Activity 

**Figure 5:**
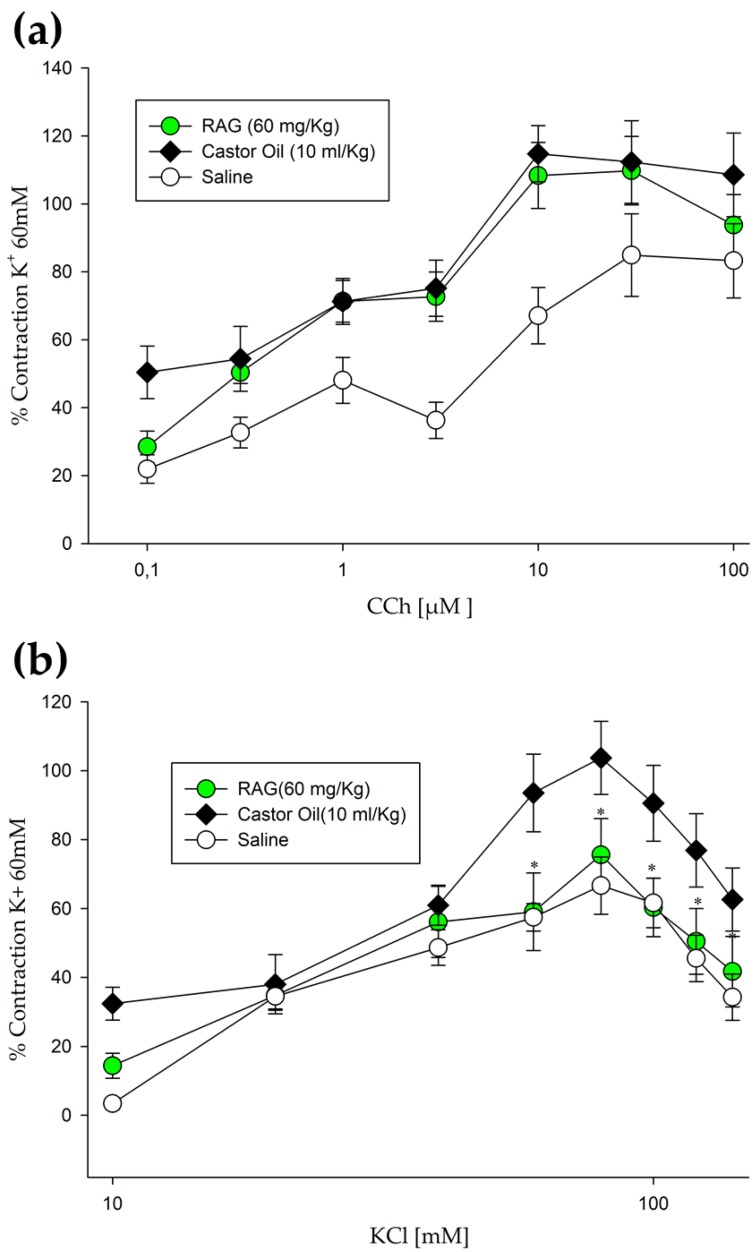
Ex vivo antispasmodic activity of RAG (60 mg/Kg). (**a**) Concentration–response curves of CCh demonstrating that oral administration of castor oil (10 mL/kg) significantly increased (*p* < 0.05) the contractions in intestinal preparations (jejunum-ileum). However, tissues from animals treated with RAG did not show responses that differed from those challenged with castor oil. (**b**) Intestinal preparations from mice pretreated with RAG 1 h before the castor oil challenge showed significantly lower contractile responses (*p* < 0.05) in response to increasing KCl concentrations. Statistics: two-way ANOVA with Holm–Sidak multiple comparison test. Abbreviations: RAG = red angico gum; CCh = carbachol.

To explore the antispasmodic activity of RAG and the possible underlying mechanism of its antidiarrheal effect, the animals were treated with RAG (60 mg/kg, p.o.) and intestinal preparations were collected for ex vivo evaluation. Oral administration of castor oil (CO, 10 mL/kg) significantly increased (*p* < 0.05, two-way ANOVA) the contractions in intestinal preparations (jejunum-ileum) in response to carbachol (CCh; 114. 7 ± 8.2% to 10 μmol/L carbachol) (Figure 5a) and KCl (103. 7 ± 10.6% to 80 mM KCl) (Figure 5b) when compared to intestinal preparations of mice treated with saline only, which showed a maximum contraction of 67.1 ± 8.3% (n = 10) and 66.6 ± 8.2% (n = 8), respectively. Intestinal preparations from mice pretreated with RAG (60 mg/kg) 1 h before CO challenge showed significantly lower contractile responses (*p* < 0.05, Holm-Sidak test) in response to KCl, which showed a maximal contraction of 75.5 ± 10.5% at 80 mM KCl (n = 8) (Figure 5b). However, tissues from animals treated with RAG and stimulated by CCh did not show responses that differed from those challenged with CO (108. 3 ± 9.7% at 10 μmol/L carbachol) (Figure 5a). The literature describes that isotonic high K^+^ concentration solutions (>30 mM K^+^) are known to cause smooth muscle contraction through the opening of voltage-dependent L-type Ca^2+^ channels, thus allowing an influx of extracellular Ca^2+^ [68], resulting in contraction. Thus, a substance that inhibits high K^+^ contractions could also be considered as a blocker of Ca^2+^ influx [69]. Considering the chemical properties of RAG, it could be possible that this polysaccharide may physically block the influx of extracellular Ca^2+^, perhaps by forming a film in the mucosa, offering topical protection. This has previously been shown to be the case in studies conducted with cashew gum, a biopolymer obtained from the trunk exudate of *Anacardium occidentale* L. and chemically and structurally similar to RAG, which provided topical protection against an “aggressive” acid solution in laryngeal and esophageal mucosa [70,71,72]. This can be further corroborated by the fact that RAG inhibited the transit of charcoal marker in the normal gastrointestinal transit model, as described above. 

### 2.3. RAG Exerts Antisecretory Activity Against Bacterial Toxins

#### 2.3.1. Evaluation of RAG on Cholera Toxin-induced Fluid Secretion in Intestinal Closed Loops

**Figure 6:**
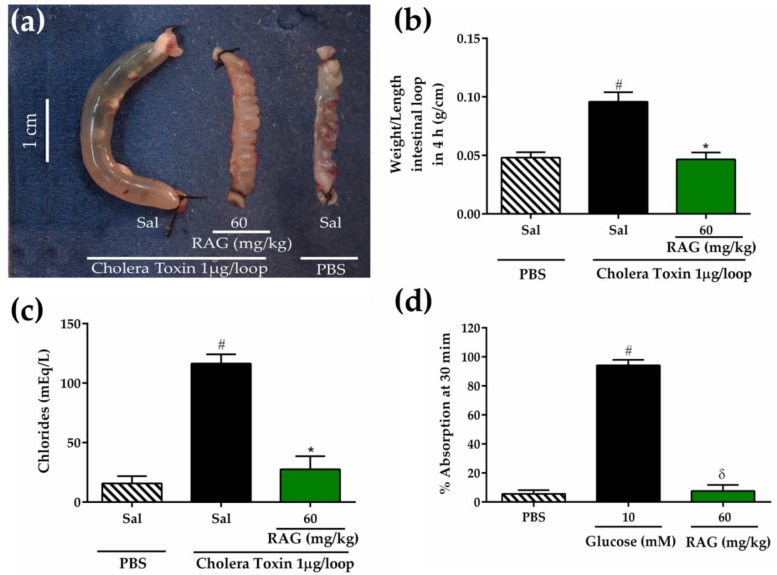
Inhibitory effects of RAG on CT-induced fluid secretion in the intestinal loops. (**a**) Intestinal fluid secretion accumulation was measured at 4 h after inoculation of CT (1 μg/loop) into an intestinal loop. (**b**) Fluid secretion was measured indirectly by the ratio of the loop weight/length. (**c**) The levels of Cl- were measured in the intestinal contents of isolated loops inoculated with CT. (**d**) Intestinal fluid absorption was measured in isolated loops 30 min after injection with RAG (60 mg/kg), PBS, or glucose (10 mM). The fluid absorption was calculated by the difference between full and empty loops and measured as the weight/length ratio. A minimum of five animals were used per group. The results are expressed as the mean ± S.E.M. * *p* < 0.05 vs. the CT group; # *p* < 0.001 vs. the PBS group; δ *p* < 0.05 vs. the glucose group. Statistics: one-way ANOVA with Tukey post-hoc test. Abbreviations: RAG = red angico gum; CT = Cholera Toxin.

The antisecretory effect of RAG was examined using a mouse intestinal closed-loop model. Figure 6a,b shows that intraluminal injection of RAG (60 mg/kg) decreased CT-induced intestinal fluid secretion by 0.095 ± 0.008 g/cm for the CT-treated group versus 0.046 ± 0.005 g/cm for the RAG treated group (*p* < 0.05) after 4 hours. These early results show that RAG is able to reduce fluid production after four hours of cholera toxin-induced diarrhea, as reported by [57], [73], and [74], proving the action of different types of natural products against cholera toxin-induced diarrhea. 

In order for cholera-secreting diarrhea to be installed, two important factors stand out for the full pathogenicity caused by *Vibrio cholerae*: bacterial pilus production [75] and cholera toxin (CT), which is responsible for the whole chain of events linked to the diarrheal condition it produces, from its binding to the GM1 receptor, culminating in the intense fluid and chloride ions loss characteristic of this disease [76]. In our study, we confirmed that CT (116.50 ± 7.76 mEq/L) significantly increased the levels of chloride ions in the intestinal loop compared with the PBS group (15.73 ± 6.13 mEq/L). However, treatment with RAG significantly (*p* < 0.05) inhibited CT-induced Cl^−^ secretion (27.62 ± 10.95 mEq/L) as shown in Figure 6c. When an intraluminal injection of RAG was made, which strongly inhibited cholera toxin induced fluid secretion, there was no significant change in intestinal fluid absorption (Figure 6d). This indicates that the inhibition of CT stimulated intestinal fluid secretion after RAG treatment is likely mediated by another mechanism. Similar results have been previously reported by Araújo et al. [32] for cashew gum.

#### 2.3.2. GM1 Ganglioside-Dependent ELISA

**Figure 7:**
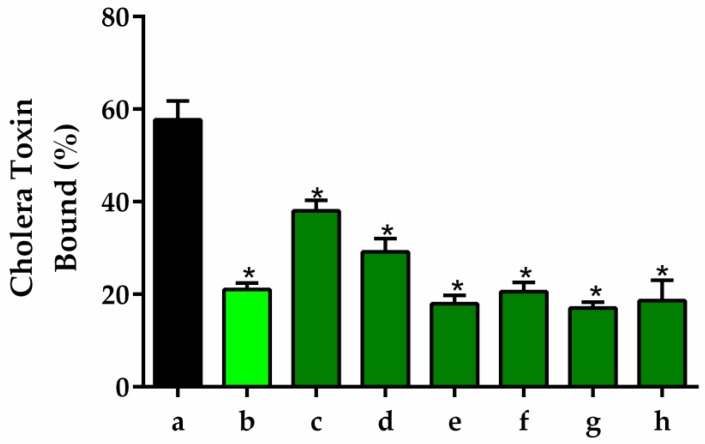
Effects of RAG on cholera toxin (CT) and GM1 binding. CT (100 ng) was incubated alone (column a) or with 1, 10, 50, 100, 300, or 500, µg/mL of RAG (columns c to h, respectively), and the amount of CT was estimated by GM1 ELISA. GM1-coated wells were preincubated with RAG and washed, and then 100 ng of CT was added to the wells and the amount of CT was estimated by ELISA (column b). Values obtained for 100 ng of CT were taken as 100% binding. Data shown are means ± SEM from three independent experiments performed under similar conditions. * *p* < 0.001 vs. column a. Statistics: one-way ANOVA with Tukey post-hoc test.

Cholera toxin consists of two A subunits and five B subunits, essential for binding to monosialoganglioside 1 (GM1) receptors on the surface of enterocytes in mucosa [77]. After toxin binding, a cascade involving G proteins, adenylyl cyclase, cAMP, and protein kinase A (PKA) promotes the efflux of Cl^−^ with an associated massive loss of water [76]. To examine the possibility that RAG prevents the CT-GM1 binding, which could be due to interaction of RAG with CT or the GM1 receptor, ELISA was carried out initially with GM1 incubated with RAG, washed, and then followed by incubation with cholera toxin (CT; 100 ng/mL). The results show that prior incubation of GM1 with RAG (100 μg/mL) significantly decreased the detection of CT by ELISA, suggesting that GM1 interacts with RAG (Figure 7, column b). This result is corroborated by the observation that the levels of CT decreased in a dose-dependent manner in parallel with the increase (1–500 µg/mL) in concentration of RAG (Figure 7, columns c to h). Taken together, these results suggest that RAG interacts with the GM1 receptor to prevent CT-GM1 binding. 

Some in vitro studies have shown that several compounds can act as analogue receptors or anti-adhesive agents for GM1, such as galactose derivatives [78], phenyl-ring containing galactose derivatives [79], sialyloligosaccharides (SOS) [80,81], sulfated polysaccharides [45,46,47,57], and biopolymers extracted from the trunk exudates of trees [32], and they may offer a promising alternative to conventional treatment strategies. All these compounds mentioned are structurally similar and contain galactose, and similarly, the chemical characterization of RAG reported that it contains 24.1% galactose in % weight [26,82]. Thus, it is possible that these regions could somehow interact with the GM1 binding site to prevent CT binding, and reducing the pathophysiological manifestations of cholera. 

#### 2.3.3. Evaluation of RAG in Secretory Diarrhea Induced by ETEC

CT shares a homologous amino acid sequence with heat-labile toxin (LT) produced by ETEC [83]. Moreover, this toxin binds to GM1 receptors in the gut epithelium just like CT, initiating the same cell signaling cascade observed in the pathophysiology of cholera [84]. Due to the structural and mechanical similarities of these toxins, and the effectiveness of RAG in CT-induced diarrhea, the action of this biopolymer was also investigated in a diarrhea model induced by ETEC.

Our results show that RAG treatment significantly (*p* < 0.05) decreased the total feces and fecal weight when compared to the positive control group (saline + *E. coli*). The percentage of inhibition of diarrhea in the group treated with RAG was 51.61% and in the group treated with gentamicin was 62.98% (Table 5). Additionally, on the third day, it was observed that RAG or gentamicin treatment significantly prevented weight loss in the animals (1.6 and 2.1 g, respectively) when compared to the positive control group (saline + *E. coli*, 5.4 g) (Table 6).

**Figure 8:**
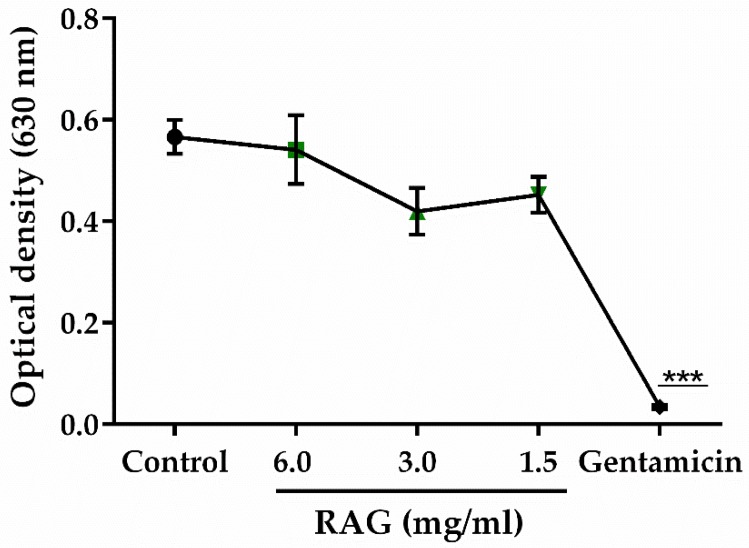
Antibacterial activity test. All tested doses of RAG (0.05–6 mg/mL) showed absence of inhibiting growth of *E. coli*. The graph shows only the doses of 6, 3, and 1.5 mg/mL of RAG. The standard antimicrobial drug, gentamicin, shows a significant inhibition on E. coli growth. Data shown are means ± SEM from three independent experiments performed under similar conditions. *** *p* < 0.001 vs. control. Statistics: one-way ANOVA with Dunnett’s multiple comparisons test.

To investigate whether the antisecretory effect of RAG against ETEC was due to a mechanism similar to that proposed in CT-induced diarrhea, blocking binding to the GM1 receptor, the minimum inhibitory concentration (MIC) of RAG against *Escherichia coli* ATCC 25922 (at a concentration of 5 × 10^5^ CFU/mL) was determined. The results obtained show no inhibition of growth at the tested doses (0.05–6 mg/mL) (Figure 8). Thus, these results suggest that these concentrations of RAG do not exert antibacterial activity against *E. coli*. 

Similar results have been observed by Quelemes et al. [40] where the use of cashew gum, which is structurally similar to RAG, did not inhibit bacterial growth within the range of concentrations tested. It is known that molecular weight, charge density, and/or the distribution of the polysaccharides play an important role in their biological activity [85]. Therefore, the absence of antimicrobial activity by RAG could be attributed to the repulsion between the charge of the molecule (zeta potential −24.50 ± 0.9 mV) and the cell wall of the bacterium, where the zeta potential can range from −10 to −90 mV [86]. Another mechanism may include enzyme production by the bacterium which can be capable of removing the polysaccharides from the cell surface [87]. Such a proposition can be corroborated by the fact that the quaternization of cashew gum derivatives, with cationic composition, show antimicrobial activity against the selected bacteria [40].

### 2.4. Acute Oral Toxicity Test

In this study, to evaluate the acute toxicity of RAG, we followed the recommendations of OECD Guideline 423 (2001). According to this guideline, when information available in the literature about the substance suggests that toxicity or mortality is unlikely even at high doses, like polysaccharides, the limit test can be conducted [47,88]. The limit dose of 2000 mg/kg did not cause death or any toxic signs in treated mice. All experimental animals were normal throughout the study and survived until the last day of analysis. No significant differences were observed between groups in terms of clinical or behavioral changes, hematological parameters, water/food consumption, and excreta produced (diarrhea and/or constipation) after 14 consecutive days (data not shown).

The response of the body to toxic substances is also manifested by physiological changes, which can be detected by changes in certain enzymes and plasma proteins. The plasma levels of the enzymes aspartate transaminase (AST), alanine transaminase (ALT), total proteins (useful in determining the extent hepatic injury), and urea and creatinine (sensitive indicators of renal function) were not significantly different from control groups (Table 7). Because the animals from the vivarium of the Federal University of Piauí do not have a serum standard for these dosages, they were compared to the animals from the central vivarium of the Federal University of Ceará.

**Figure 9:**
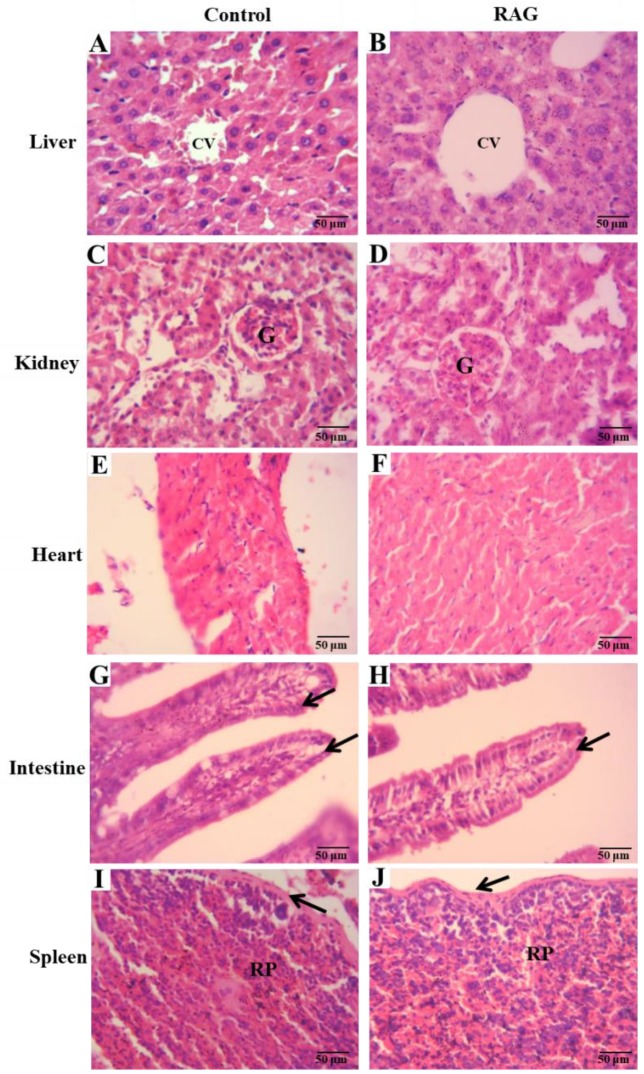
Micrographic characterization of histopathological changes in the organs 14 days after single oral administration of RAG (2000 mg/kg). (**A**) Indicates the hepatic tissue of the saline group (control) and (**B**) the RAG group, both groups, did not show histological changes. CV, central vein; Renal cortex of: (**C**) control and (**D**) RAG groups, with both groups presenting typical renal corpuscle with glomerulus (**G**) and normal capsular space surrounded by Bowman’s capsule, around the corpuscles are the regular proximal convoluted and distal convoluted tubules; Heart: (**E**) control group and (**F**) RAG group demonstrating normal heart tissue, no necrosis nor neutrophil infiltration; Intestine: (**G**) control group and (**H**) RAG group illustrating typical mucosa, absence of necrosis, and neutrophil infiltration with normal characteristics of epithelial cells (arrows) and goblet cells. Spleen: (**I**) control group and (**J**) RAG group demonstrating habitual architecture, red pulp [RP] and capsule of connective tissue indicated by arrows. Hematoxylin and eosin staining in (**A**–**J**); original magnification 150×.

Furthermore, the assessment of the organs such as the liver, kidney, heart, gut and spleen, which are sensitive to and important in the metabolism and excretion of xenobiotics, did not reveal any abnormalities in their gross examinations or difference in their mean weights either in treated or in control groups (Table 7). Corroborating these findings, the histological analysis of these organs also revealed no pathological changes between groups (Figure 9). Similarly, Schmitt et al. [89] and Nicolau et al. [72] did not observe significant changes in these parameters in toxicologic evaluation of acacia gum or cashew gum, respectively. Together, these results, according to the OECD Guideline 423 (2001), suggest low toxicity of RAG, which can be classified as category 5 (substance with LD50 higher than 2000 mg/kg) of the globally harmonized system (GHS). According to the protocol, the next dose to be tested would be 5000 mg/kg. However, the study of this dose is only recommended in exceptional cases [90]. 

## 3. Materials and Methods 

### 3.1. Chemicals

The following materials were obtained from commercial sources: castor oil, prostaglandin E2, charcoal, atropine, carbachol (CCh), Splittgerber’s reagent, cholera toxin, monosialoganglioside-GM1, and 3,3’,5’,50-Tetramethylbenzidine, from Sigma-Aldrich, Inc. (St Louis, MO, USA); bethanechol chloride, loperamide hydrochloride, and naloxone hydrochloride from Janssen-Cilag Pharmaceutics LTDA, Brazil and CRISTÁLIA Pharmaceutical Chemicals products LTDA, Brazil; Xylazine hydrochloride and ketamine hydrochloride, were obtained from Syntec (Cotia, SP, Brazil). All other chemicals used were of analytical grade and obtained from standard commercial suppliers. All drugs were dissolved in saline or phosphate-buffered saline (PBS).

### 3.2. Plant Material

Crude polysaccharide samples of RAG were collected in 2016 by the Biotechnology and Biodiversity Center Research–BIOTEC, Federal University of the Parnaíba Delta, Parnaíba-PI, Brazil, from the trunks of native red angico trees (*A. colubrina* var. cebil (Griseb.) Altschul) in the municipality of Simplício Mendes, Piauí, Brazil. Additionally, a voucher of this specimen was deposited in herbarium of the Parnaíba Delta (HDELTA), Parnaíba-PI, Brazil, (nº 3618).

### 3.3. Extraction and Purification of RAG

RAG was purified as a sodium salt using a previously described method [91]. Crude samples of exudate were selected and dissolved in distilled water at 25 °C for 24 h, providing a 5% (w/v) solution. The pH of the solution was adjusted to approximately 7.0 by addition of diluted aqueous NaOH. For precipitation, RAG was added to a beaker containing 30 mL of ethanol during a 24-hour refrigeration period. The precipitate formed on the bottom was separated from the liquid and washed twice with ethanol to remove any water and impurities. The product was macerated and washed again with ethanol, followed by acetone to further remove any impurities and water. The washed precipitate was dried and macerated with exposure to frequent hot air flow until the gum was obtained [82].

### 3.4. Characterization of RAG by Infrared Spectroscopy, Zeta Potential, Elemental Analysis, Molar Weight, Solubility and Impurity Determination

The Fourier transform infrared spectra (FT-IR) of the biopolymer were recorded with a Shimadzu IR spectrophotometer (Affinity-1S-ATR-attenuated Total Reflectance, ZnS crystal, model 8300) between 700 and 4000 cm^−1^. Samples were analyzed by absorbance in attenuated total reflection (ATR). All analyses were performed with 45 scans on zinc selenide crystal for the identification of the functional groups present in RAG. Zeta potential was measured using the Malvern Zetasizer Nano ZS90 with the following settings: dispersant: water, temperature: 25 °C, calibration time: 2 min, number of scans: 173, scan time: 20 min, and a 10 mg/mL aqueous solution was used. The measurements were carried out in triplicate to determine biopolymer load. The elemental analyses for carbon, hydrogen, and nitrogen composition were performed using a Perkin Elmer 2400 series CHNS analyzer with a thermal conductivity detector. 

The molar mass distribution was determined by the chromatography of gel permeation using the Shimadzu LC-20AD coupled to a refractive index (RID-10A). For the analysis, we used a linear polysep column, 300 × 7.8 mm, using 0.1 mol/L NaNO_3_ (aq) as the eluent. Solubility was measured at 30 °C and a flow rate of 1 mL/min with an injected sample volume of 50 µL. A mass of 0.1 g RAG was applied to 1 mL distilled water at different pHs (2, 7, and 8), adjusted with HCl and 0.5 mol/L NaOH, and allowed to stir for 12 hours, the samples then underwent ultrasound for 15 min to aid in the solubilization process. Subsequently, centrifugation was performed for 10 min at 10,000 rpm for separation and precipitation. At weighing, samples were dried using vacuum centrifugation. The degree of impurity was determined by adding 0.1 g RAG to 10 mL acetone and stirring for 3 h, followed by centrifugation for 10 min at 3600 rpm. Samples were then dried and the supernatant centrifuged under vacuum for 5 h. All dry content was weighed.

### 3.5. Evaluation of Antidiarrheal Activity of RAG

#### 3.5.1. Animals and Ethical Considerations

Mice (Swiss strain, 25–30 g) of both sexes were obtained from the Central Vivarium of Federal University do Piauí, Teresina, Piauí, Brazil. All animals were kept and maintained in cages under laboratory conditions at a temperature of 23 ± 1 °C under a 12 h light/12 h dark cycle with free access to food (standard pellet diet) and drinking water ad libitum. The animals were divided into groups of 6–8 animals per group for treatment with RAG, the reference drug, or saline-treated control. The animals were deprived of food for 18–24 h before the experiments, with free access to water. All experimental procedures and protocols used in this study were approved by the Ethics Committee in Research of the Federal University of Piauí (protocol no. 068/2014). This study was performed in accordance with the recommendations in the Guide for the Care and Use of Laboratory Animals (National Institutes of Health, Bethesda, MD, USA) and the ARRIVE (Animal Research: Reporting of In Vivo Experiments) Guidelines.

#### 3.5.2. Castor Oil-Induced Diarrhea

Castor oil was used to induce diarrhea according to a previously described method [64], with some modifications. Mice were randomly allocated to groups (6–8 mice per group) and starved for 18 h before the experiment with free access to water. The treatment groups were allocated as follows: Saline, RAG 30, 60 and 120 mg/kg, loperamide (a standard antidiarrheal agent) 5 mg/kg, and vehicle only. All test substances were administered by gavage. After 1 h, diarrhea was induced in the experimental groups by administering castor oil (10 mL/kg p.o.), the vehicle group received saline only. Immediately after administration, the animals were placed in cages lined with adsorbent paper and were observed for 3 h to detect the presence of characteristic diarrheal droppings defined as watery (wet), unformed stools. The total mass of fecal output (mg) and the total number of diarrheic stools (mg) excreted by each group in that period of time were monitored. The severity of the castor-oil induced diarrhea was scored based on stool consistency and recorded. Scores were assigned using a previously described method [63], which is as follows: normal stool (or lack of diarrhea) = 0, semi-solid stool = 1, pasty stool/feces in small/moderate amount = 2, and watery stool/feces in large amount = 3. The efficacy of each treatment was expressed as percent inhibition (%) of diarrhea compared to the control group result expressed as 100%. The percent inhibition of defecation and diarrhea was calculated by:

% Inhibition of defecation = [(A − B)/A] × 100(1)

where A represents the mean mass of defecation caused by castor oil and B represents the mean mass of defecation after treatment with drug or RAG.

#### 3.5.3. Castor Oil-Induced Intestinal Fluid Accumulation (Enteropooling)

Measurement of the castor oil-induced enteropooling was carried out according to the method previously described by Robert et al. [92] with some modifications. Mice were deprived of food for 18 h before the experiment and randomly allocated into groups of 6–8 rats per group. Initially the control group received saline while the experimental groups were pre-treated with RAG (30, 60, and 120 mg/kg) or loperamide (5 mg/kg). After 1 h, diarrhea was induced in all groups with castor oil (10 mL/kg p.o.). All test substances were administered by gavage. After 3 h, the animals were euthanized using a combination of xylazine hydrochloride and ketamine, the small intestine from the pylorus to the caecum was then isolated, and the intestinal contents were measured in a graduated tube. The efficacy of each treatment was expressed as percent inhibition (%) of the volume of intestinal fluid and was calculated by:

% Inhibition of the volume of intestinal fluid = [(A − B)/A] × 100,(2)

where A represents the mean of the volume of fluid after castor oil administration alone; B represents the mean volume of fluid after pretreatment with Castor oil and treatment with drug or RAG.

#### 3.5.4. PGE_2_-Induced Enteropooling

Prostaglandin E2 (PGE_2_) was used to induce intestinal fluid accumulation according to the method previously described by Mukherjee et al. [93], with some modifications. Mice were deprived of food for 18–24 h before the experiment. The control group received saline orally and the experimental groups received RAG (60 mg/kg) or loperamide (5 mg/kg) by gavage. Immediately after drug administration, intestinal fluid accumulation was induced in groups by oral administration of PGE2 (100 μg/kg; Sigma Aldrich, USA) to each mouse. The vehicle group received saline only. Thirty minutes after administration of PGE_2_, mice were euthanized, and the small intestine from the pylorus to the caecum was resected. The content was collected in a graduated tube and the volume was measured. The efficacy of each treatment was expressed as percent inhibition (%) of the volume of intestinal fluid and was calculated identical to the castor oil-induced enteropooling model described above.

#### 3.5.5. Assessment of Na+/K+-ATPase Activity

Mice were deprived of food for 18 h with free access to water. Castor oil was used to induce diarrhea as described above. The control group received saline, while experimental groups received RAG (60 mg/kg, p.o.) or loperamide (5 mg/kg, p.o.). After 1 h of pretreatment, castor oil (10 mL/kg p.o.) was administered to the experimental groups and the vehicle group received saline only. After 3 h, the mice were euthanized, laparatomized, and the small intestine (from the pylorus to the caecum) removed to evaluate Na+/K+-ATPase activity, as previously described [94]. The activity of Na+/K+-ATPase was standardized to the protein concentration in the samples of the small intestine tissues using standard procedures, according to the manufacturers’ instructions (Labtest Diagnostica, Lagoa Santa, Brazil). Thereafter, the activity of Na+/K+-ATPase was calculated using the following equation:

Specific activity (µmole Pi•mg protein^−1^ h^−1^) = [Pi] × 2 × dilution factor/1000 × protein concentration (mg/mL) (3)

where [Pi] represents the concentration of inorganic phosphate in nmoles (as obtained from the calibration curve, 2 represents the factor required to obtain the amount of Pi released per h, and 1000 represents the factor introduced to convert the Pi released to µmoles.

#### 3.5.6. Gastrointestinal Transit Test (Charcoal Meal)

The effect of RAG on gastrointestinal transit was evaluated using a previously described procedure, whereby charcoal meal is used as a marker of the distance traveled [63]. The experiment was performed in four stages separately, aiming to evaluate the possible involvement of opioid receptors or anticholinergic activity, on the effect of RAG under the inhibition of gastrointestinal transit. Initially, mice were randomly divided into groups of six animals and fasted for 18 h before the respective tests, but with free access to water. 

In the first series of experiments the effect of RAG on the reduction of gastrointestinal transit was evaluated using the castor oil-induced diarrhea model. All animals received castor oil (10 mL/kg, p.o.) to induce diarrhea and after 1 h were treated orally with saline (2.5 mL/kg), RAG (60 mg/kg) or loperamide (5 mg/kg). One hour after drug administration, all animals orally received a charcoal meal (0.2 mL of 10% activated charcoal suspended in 5% gum acacia) by gavage. After 20 minutes, the animals were euthanized. The distance covered by the charcoal meal in the intestine from the pylorus to the caecum was measured and expressed as a percentage of distance covered where:

% Distance covered by marker = [A/B] × 100(4)

where A represents the mean of the distance traveled by the charcoal meal and B represents the mean total length of intestine.

In the second series of experiments, to examine the involvement of opioid receptors on the effect of RAG under reduced gastrointestinal motility, all mice were pre-treated with naloxone (2 mg/kg, s.c., opioid antagonist). After 30 minutes, the animals were treated with saline (2.5 mL/kg), RAG (60 mg/kg) or loperamide (5 mg/kg, opioid agonist). The steps following were performed as described above.

In the third series of experiments, to evaluate a possible anticholinergic activity of RAG in the reduction of gastrointestinal transit, was performed the gastrointestinal transit model stimulated by bethanechol, a cholinergic agonist of muscarinic type 3 receptors [95]. Mice were pre-treated orally with saline (2.5 mL/kg), RAG (60 mg/kg), or atropine (3 mg/kg, s.c., cholinergic antagonist). Thirty minutes later, the animals received bethanechol (3 m/kg, i.p.), and the negative control group received saline. One hour later, all animals orally received a charcoal meal (0.2 mL of 10% activated charcoal suspended in 5% acacia gum). Twenty minutes later, the mice were euthanized and the distance covered by the marker in the intestine, from the pylorus to the cecum, was measured as described above.

Additionally, to assess the effect of RAG on normal transit, mice were pre-treated orally with saline (2.5 mL/kg), RAG (60 mg/kg) or loperamide (5 mg/kg). One hour later, all animals orally received a charcoal meal by gavage. Twenty minutes later, mice were euthanized and the distance covered by the marker was measured as previously described. 

#### 3.5.7. Contraction Induction of Isolated Mouse Jejunum-Ileum Ex Vivo

Evaluation of the effect of RAG on inducing contractions in isolated jejunum-ileum ex vivo was performed using previously described methods [96], with some modifications. Mice were randomly allocated to groups and starved for 18 h, before the experiment, with free access to water. The treatment groups were as allocated as follows: saline, RAG (60 mg/kg) and vehicle only. After 1 h, diarrhea was induced in the experimental groups by administering castor oil (10 mL/kg, p.o.), the vehicle group received saline only. All test substances were administered by gavage. After 3 h, the mice were euthanized and gut preparations (jejunum- ileum) were obtained. The tissues were isolated after opening of the abdomen. Elongated segments (2–3 cm) were placed in a tissue bath (10 mL) comprising of Tyrode’s solution, kept at normal body temperature of 37 °C with constant aeration, a mixture of O_2_ and CO_2_ (Carbogen/95% + 5%). Tyrode’s solution (mM) comprises potassium chloride (KCl) 2.68, NaCl 136.9, MgCl_2_ 1.05, NaHCO_3_ 11.90, NaH_2_PO_4_ 0.42, CaCl_2_ 1.8, and glucose 5.55 (pH 7.4). A preload of 1 g was used and contractions in the intestinal tissue were noted using a force displacement transducer (MLT0015) attached with bridge amplifier and Power Lab 4/25 data acquisition system coupled to a computer running Lab Chart 6 software (AD Instrument, Australia). The tissues were allowed to equilibrate for a period of 30 min, and then were repeatedly stimulated with 60 mM/L K^+^ to evaluate the tissue viability. Preparations without reproducible contractions were discarded. These contractions served as a reference and allowed the comparison between different tissues, and the results presented in this study are generally expressed as a percentage of the last K^+^-induced contraction. Contractile responses to carbachol (CCh; 0.1, 0.3, 1, 3, 10, 30, and 100 µM) and KCl (10, 20, 30, 40, 60, 80, 100, 120, and 140 mM) were recorded at 5-min intervals in animal samples, pretreated with or without RAG (60 mg/kg). The relaxant effects of RAG were observed when the spontaneous contractions of the preparation showed percent change, recorded directly before and after the adding of contraction inducing substances.

#### 3.5.8. Cholera Toxin-Induced Secretory Diarrhea 

The inhibitory effect of RAG on intestinal fluid secretion induced by cholera toxin (CT) inoculation in mice-intestinal closed loops was measured using a previously described method [97]. The animals were given access to water but not food for 24 h before the experiment. Mice in the negative group and positive control group were pretreated orally with saline (2.5 mL/kg), while the test group (group 3) received RAG (60 mg/kg). After 1 h, the animals were anesthetized intraperitoneally with a combination of xylazine hydrochloride (5 mg/kg) and ketamine (60 mg/kg) and a median laparotomy was performed, where an abdominal incision was made to expose the small intestine, and closed jejunal loops (2–3 cm) were isolated using sutures. In this procedure, the body temperature was maintained during surgery at 36–38 °C using a heating pad. Intestinal loops were inoculated with 100 μL of phosphate-buffered saline (PBS) for negative group, and CT dissolved in PBS at a dose of 1 μg/loop for control and test groups. The abdominal incision was closed with sutures, and the animals were allowed to recover from anesthesia. At four hours, the mice were euthanized, and the closed loops were rapidly removed from the abdominal cavity. The fluid secretion was measured indirectly as the ratio of loop weight/length expressed in g/cm. The intestinal contents accumulated in each closed loop were collected separately to measure the concentration of chloride (Cl^-^) ions according to the manufacturer’s instructions (Labtest, Lagoa Santa, Minas Gerais, Brazil). 

#### 3.5.9. Intestinal Fluid Absorption in Closed Loops

Initially, mice were fasted for 24 h before experiment. Jejunal loops were isolated as described in the previous section. For intestinal fluid absorption studies, loops were injected with 200 µL of PBS with or without 10 mM glucose or PBS containing RAG (60 mg/kg p.o.). The intestinal loops were returned to the abdominal cavity and the laparotomy was closed with sutures. Thirty minutes after inoculation, the intestinal loops were removed, and the percentage of fluid absorption was measured indirectly as the loop weight/length ratio.

#### 3.5.10. GM1 Ganglioside-Dependent ELISA

Wells containing monosialoganglioside 1 (GM1) with or without different concentrations of RAG were used for estimation of CT by GM1 ELISA. Samples containing cholera toxin (CT) with or without different concentrations of RAG (1 to 500 μg/mL) were serially diluted and added to microtiter plates containing immobilized GM1. ELISA was carried out as previously described by Saha et al. [98]. 

#### 3.5.11. Enterotoxigenic Escherichia Coli- Induced Secretory Diarrhea

Enterotoxigenic *Escherichia coli* (ETEC) cell suspension was used to induce diarrhea according to previously described methods by Bisson et al. [99], with modifications. Mice were randomly allocated into groups with six animals per group and acclimated for 7 days before treatment. The animals were given access to water but not food 18 h before the experiment. Negative and positive controls received saline while the antibiotic group received gentamicin (8 mg/kg p.o.). The test group received RAG (60 mg/kg p.o.) for three days. ETEC was obtained from the Department of Microbiology Laboratory, Federal University of Ceará, Brazil. A cell suspension of ETEC was transferred to assay tubes containing NaCl sterile solution, and bacterial cultures were adjusted to a cell density of 2 × 10^8^ colony forming units per mL (CFU/mL) by optical density (O.D.) measurements using the spectrophotometer. Diarrhea was induced in all groups except in the negative control group by single oral administration (5 mL/kg) of ETEC solution incubated at 37 °C. The negative group received a single oral dose (5 mL/kg) of physiological saline (vehicle) warmed at 37 °C. After treatment, the animals were placed in cages lined with absorbent material to detect the amount of diarrheal stool. The total amount of stool (g) and the total amount of diarrheal stool (g) excreted by each group were monitored for three days. The effect of each treatment was expressed as the percent inhibition of diarrhea, with the value for the control group set to 100%.

#### 3.5.12. Test of the Antibacterial Activity of RAG

The minimum inhibitory concentration (MIC) of RAG against *Escherichia coli* ATCC 25922 (at a concentration of 5 × 10^5^ CFU/mL) was determined by microdilution in Mueller Hinton broth (BD Difco^™^) with concentrations of RAG ranging from 0.05–6 mg/mL. The culture was placed in 96-well micro titer wells which were incubated at 35 ± 2 °C for 24 h under aerobic conditions, the growth was measured at 630 nm (OD630) using a microplate reader (Bioeasy). The MIC was defined as the lowest concentration that gives a close to zero absorbance reading (OD < 0.05). This test was carried out in triplicate and gentamicin (8–512 μg/mL) was used as the standard antibacterial agent [100,101]. 

### 3.6. Acute Toxicity Study of RAG

For the safety assessment of RAG, the acute toxic class method described by the Organization for Economic Cooperation and Development (OECD) (Guideline 423/2001) [90] was performed. Female Swiss mice (three per group) were used for these procedures. Animals in the test group orally received a dose of RAG (2000 mg/kg) dissolved in saline and the control group received only saline.

#### 3.6.1. Clinical Observation

All animals were observed after administration (15 min, 30 min, and 1 h) and periodically during the first 24 h, with special attention given during the first 4 h, and daily thereafter, for 14 days at scheduled times. Any symptoms of illness or abnormal behavior were taken into account. Parameters of toxic signs (Hippocratic screening) were studied according to OECD guideline 423 [52], which includes tremors, salivation, convulsions, lethargy, diarrhea, sleep, and coma. Skin and fur, eyes and mucous membranes, respiratory, circulatory, autonomic and central nervous systems, somatomotor activity, and behavior patterns were also evaluated. Furthermore, the body weight of the animals, consumption of water and feed and production of excreta was monitored throughout the study period.

#### 3.6.2. Biochemical and Hematological Parameters

At the end of monitoring for toxic signs, the 14th day, all animals were anaesthetized with a combination of xylazine hydrochloride (5 mg/kg, i.p.) and ketamine (60 mg/kg, i.p.). Blood samples were then collected by cardiac puncture and stored in tubes with ethylenediaminetetraacetic acid (EDTA) for the hematological analysis. Biochemistry analysis was then performed on the plasma using the Semi-automatic biochemical analyzer TEKNA (Labtest Diagnosis, São Paulo, Brazil) for the following parameters: aspartate aminotransferase (AST), alanine aminotransferase (ALT), total proteins, creatinine, and urea, according to the manufacturer’s specifications.

#### 3.6.3. Organ Weights and Histological Analysis

On the 14th day, after cardiac puncture and subsequent euthanasia, mice were then laparatomized and organs (liver, kidney, heart, spleen and small intestine) were removed and weighed. The relative weight of each organ was calculated using the following expression: organ weight/body weight after treatment ×100. For histological analysis, a portion of 3 cm of the small intestine (jejunum) and organs mentioned above were fixed in alcohol, cleared in xylene, and embedded in paraffin. The blocks were in 10% buffered formalin. After fixing, the tissues were dehydrated and sliced into 5-µm sections, stained with hematoxylin-eosin (HE), and observed under a light microscope.

### 3.7. Data and Statistical Analysis

Data are presented as the mean (±SEM) for the animals in each group (n = 6–8). Results from saline-treated control mice were used as baseline values. In all cases, the results obtained from RAG- or reference drug-treated test groups were compared with those obtained from saline-treated controls. The in vitro test data represent the mean ± SEM from three independent triplicate experiments. Statistical tests were performed using GraphPad Prism (version 6.0) software. The statistical significance of the differences between groups was determined by one-way analysis of variance (ANOVA) and the Tukey post-hoc test. To study the fluidity of feces and histological analysis, the Kruskal–Wallis nonparametric test was used, followed by Dunn’s post-hoc test for multiple comparisons. For the toxicological analysis, the differences between the control group and the test were determined by Student’s t test. Differences were considered to be significant when *p* < 0.05. To determine the significance of the in vitro results one- or two-way analysis of variance (ANOVA) was used and, when significant, was followed by the Holm–Sidak multiple comparisons test. 

## 4. Conclusions

In this study, the polysaccharide extracted from the trunk exudate of *A. colubrina* (RAG) was successfully purified and characterized with results corroborating that of the literature. FT-IR of RAG revealed a chemical identity compatible with the polysaccharides, with a molar mass of 1.89 × 10^5^ g/mol and a negative zeta potential. Furthermore, RAG showed high yield and solubility at different pHs with a low degree of impurity.

The results obtained in the biological assays suggest that the antidiarrheal activity of RAG is probably due to its ability to 1) alter fecal parameters, 2) reduce gastrointestinal motility, possibly through physical blocking of extracellular ion influx in the mucosa, and thus 3) inhibit intestinal smooth muscle contractions. Additionally, RAG displayed effective antisecretory activity against CT- and ETEC-induced diarrhea, likely due to blocking the binding of these toxins to the GM1 receptor. 

These results are substantiated by the low acute oral toxicity observed, providing scientific support for the traditional use of RAG tree exudate for the treatment of diarrhea by the local communities of northeastern Brazil. In conclusion, since there is an increasing need for new antidiarrheal treatments that do not exhibit side effects similar to that of standard drugs, further studies could evaluate other parameters involved in the antidiarrheal activity of RAG to validate its use as a novel natural antidiarrheal agent.

## Figures and Tables

**Table 1:** Chemical feature results obtained of RAG and its pH solubility.

Sample	Mean Molecular Weights (g/mol) and Polydispersity			Elemental Analysis (%)			pH Solubility (%)		
**RAG**	**Mn**	**Mw**	**Đ**	**C**	**H**	**N**	**2**	**7**	**8**
	1.79 × 10^3^	1.89 × 10^5^	1.89 × 10^1^	38.31	5.985	0.47	99 ± 0.1	98.5 ± 0.5	99.1 ± 0.1

**Table 2:** Effect of red angico gum (RAG, 30–120 mg/kg, p.o.) on castor oil-induced acute diarrhea in mice.

Treatment	Dose	Total No. of Stools (g)	Inhibition of Defecation (%)	Total Amount of Diarrheal Stool (g)	Inhibition of Diarrhea (%)
**Control**	2.5 (ml/kg)	5.45 ± 0.02	0.0	5.22 ± 0.02	0.0
**RAG**	30 (mg/kg)	3.92 ± 0.02 *	28.1	3.62 ± 0.02 *	33.71
	60 (mg/kg)	2.44 ± 0.02 *	55.3	2.03 ± 0.02 *	61.18
	120 (mg/kg)	2.14 ± 0.01 *	60.8	1.45 ± 0.02 *	73.34
**Loperamide**	5 (mg/kg)	1.28 ± 0.02 *	76.5	0.99 ± 0.02 *	77.62

Values are presented as mean ± SEM. Number of animals/group: 5–7. * *p* < 0.05 vs. control group (saline), Statistics: One-way ANOVA with Tukey post-hoc test. Loperaminde: standard antidiarrheal drug.

**Table 3:** Effect of red angico gum (RAG, 30–120 mg/kg, p.o.) on castor oil-induced intestinal fluid accumulation (enteropooling) in mice.

Treatment	Dose	Intestinal Fluid (mL)	Inhibition of Intestinal Fluid Volume (%)
**Control**	2.5 (ml/kg)	0.53 ± 0.02	0.0
**RAG**	30 (mg/kg)	0.47 ± 0.06	10.5
	60 (mg/kg)	0.23 ± 0.04 *	56.7
	120 (mg/kg)	0.21 ± 0.04 *	59.4
**Loperamide**	5 (mg/kg)	0.30 ± 0.06 *	43.6

Values are presented as mean ± SEM. Number of animals/group: 5–7. * *p* < 0.05 vs. control group (saline). Statistics: One-way ANOVA and Tukey post-hoc test.

**Table 4:** Effects of red angico gum (RAG, 60 mg/kg p.o.) on PGE_2_-induced intestinal enteropooling and fluid accumulation in mice.

Treatment	Dose	Intestinal Fluid (mL)	Inhibition of Intestinal Fluid Secretion (%)
**Saline**	2.5 (ml/kg)	0.34 ± 0.07	-
**Control (** **PGE_2_ in saline)**	100 (μg/kg)	0.73 ± 0.06 ^#^	-
**RAG**	60 (mg/kg)	0.47 ± 0.16	64.1%

Values are presented as mean ± SEM. Number of animals/group = 5. ^#^ *p* < 0.001 vs. saline. Statistics: One-way ANOVA with Tukey post-hoc test.

**Table 5:** Effects of red angico gum (RAG, 60 mg/kg, p.o.) on *Escherichia coli*-induced diarrhea in mice.

Treatment	Total Amount of Diarrheal Stool (g)			Inhibition of Diarrhea (%)
	Day 1	Day 2	Day 3	
**Saline (2.5 mL/kg)**	10.36 ± 1.67	15.41 ± 1.98	17.47 ± 1.19	-
**RAG (60 mg/kg)**	3.92 ± 1.05 *	8.04 ± 1.34 *	8.98 ± 1.52	51.61
**Gentamicin (8 mg/kg)**	3.11 ± 1.53 *	6.72 ± 0.63 *	6.19 ± 1.42 *	62.98

Evaluation was performed daily after administration of *E.coli* (day 0), single dose. Values are expressed as mean ± SEM (n = 6–7). * *p* < 0.05 when compared to the saline control group. Statistics: one-way ANOVA with Tukey post-hoc test. RAG and gentamicin were administered 30 min before *E. coli* administration (day 0) and daily for 3 days.

**Table 6:** Effects of red angico gum (RAG, 60 mg/kg, p.o.) in decreasing body weight on *Escherichia coli* induced diarrhea in mice.

Treatment	Weight of Animals (g)		Weight Loss (g)
	day 0	day 3	
**Sal (2.5 mL/kg)**	29.2 ± 3.4	31.8 ± 2.2	-
**Sal + *E. coli***	28.5 ± 2.7	23.1 ± 2.4	5.4 *
**RAG (60 mg/kg)**	28.3 ± 2.5	26.7 ± 2.1	1.6 ^#^
**Gentamicin (8 mg/kg)**	28.5 ± 2.3	26.4 ± 0.6	2.1 ^#^

Values are presented as mean ± SEM. Number of animals per group = 5–6. Statistics: one-way ANOVA with Tukey post-hoc test. * *p* < 0.05 vs. Sal. # *p* < 0.05 vs. Saline + *E.coli*.

**Table 7:** Biochemical analyses of plasma, body weight (g) and organ weight (%) of treated mice with red angico gum (RAG 2000 mg/kg, p.o.) after 14 consecutive days.

Parameter		Control	RAG
**Renal Function**	Creatinine (mg/dL)	0.53 ± 0.11	0.38 ± 0.08
	Urea (mg/dL)	26.05 ± 2.20	30.61 ± 3.32
**Liver function**	Total Proteins (g/dL)	3.53 ± 0.18	3.53 ± 0.10
	ALT (TGP) (U/L)	56.43 ± 7.74	47.04 ± 6.30
	AST (TGO) (U/L)	24.50 ± 4.05	19.40 ± 3.57
**Body weight (g)**	Before	17.60 ± 0.74	20.00 ± 0.63
	After	22.00 ± 0.63	25.60 ± 0.74
**Organ weight (%)**	Liver	4.81 ± 0.08	4.66 ± 0.10
	Heart	0.65 ± 0.07	0.48 ± 0.02
	Spleen	0.41 ± 0.04	0.53 ± 0.03
	Kidneys	1.19 ± 0.06	1.19 ± 0.06
	Small intestine	6.90 ± 0.41	5.78 ± 0.19

Values are presented as mean ± SEM. Statistics: Student’s t test. Abbreviations: AST = aspartate aminotransferase, and ALT = alanine aminotransferase. Organ weight was calculated as organ weight/body weight after treatment x 100 and is presented as mean ± SEM.
